# Bifunctional enhancement of oxygen reduction reaction activity on Ag catalysts due to water activation on LaMnO_3_ supports in alkaline media

**DOI:** 10.1038/srep13552

**Published:** 2015-08-27

**Authors:** Shin-Ae Park, Eun-Kyung Lee, Hannah Song, Yong-Tae Kim

**Affiliations:** 1 Department of Energy System, Pusan National University, Busan 609-735, Republic of Korea

## Abstract

Ag is considered to be one of the best candidates for oxygen reduction reaction electrocatalysts in alkaline media for application in various electrochemical energy devices. In this study, we demonstrate that water activation is a key factor in enhancing the ORR activity in alkaline media, unlike in acid environments. Ag supported on LaMnO_3_ having a high oxophilicity showed a markedly higher ORR activity than that on carbon with inert surfaces. Through various electrochemical tests, it was revealed that the origin of the enhanced ORR activity of Ag/LaMnO_3_ is the bifunctional effect mainly due to the water activation at the interface between Ag and LaMnO_3_. Furthermore, the ligand effect due to the charge transfer from Mn to Ag leads to the enhancement of both oxygen activation on Ag and water activation on Mn sites, and hence, an improvement in the ORR activity of Ag/LaMnO_3_. On the other hand, the strain effect based on the fine structure variation in the lattice was negligible. We therefore suggest that the employment of a co-catalyst or support with highly oxophilic nature and the maximization of the interface between catalyst and support should be considered in the design of electrocatalysts for the ORR in alkaline media.

The electrocatalysis of the oxygen reduction reaction (ORR) in alkaline media has received great attention recently because it is a rate-determining process in various electrochemical energy-storage and energy-conversion devices that use alkaline electrolytes, such as metal air batteries, zinc-air fuel cells (ZAFCs), and anion-exchange membrane fuel cells (AEMFCs)^1^. Since the ORR in alkaline media is much faster than in acid^2^,^3^,^4^, materials other than Pt, such as Ag^5^,^6^, Au^7^,^8^, Pd^9^,^10^, Ni^11^, Manganese oxide^12^,^13^, spinel^14^,^15^, and perovskite^16^,^17^, have been studied intensively in the last decade. Among them, Ag is considered to be the top candidate, because it is active enough for ORR and is cost-effective^18^,^19^. Various approaches have been used to enhance the activity of Ag, such as size control^20^,^21^, alloy formation^22^,^23^, and Ag/metal-oxide composite formation^24^,^25^.

In this study, we focused on a new approach for enhancing the activity of Ag, that is, the promotion of water activation through the employment of highly oxophilic support materials. It is widely recognized that the proton supply, which is essential for the reduction of O_2_ to H_2_O, is relatively easy in acid media owing to the nature of the electrolytes originally having the protons, whereas it is much more difficult in alkaline media, where the proton should be generated by water activation (*x*H_2_O + M → M-[OH]_*x*_ + *x*H^+^ + *x*e^−^)^26^. Efficient water activation is therefore a key factor in determining the ORR activity in alkaline media. Nevertheless, most recent research on water activation in electrocatalysis has focused on electrolyzer reactions to produce hydrogen, such as in the hydrogen evolution reaction (HER) and oxygen evolution reaction (OER) in alkaline media^27^,^28^ or OH poisoning in acid media^29^. N. M. Markovic *et al.* demonstrated that in alkaline media, the bifunctional effect from the combination of the noble metal and 3d transition metal hydr(oxy)oxide cluster having high oxophilicity was the main origin of the remarkably enhanced electrocatalytic activity for the HER and OER^27^,^28^. D. E. Ramaker *et al.* investigated the effect of water activation due to the oxophilicity of the 3d transition metal for alloying on OH poisoning through X-ray absorption studies, and concluded that the ORR activity in acid media was correlated directly with the water activation to generate OH poisoning on the surfaces. On the other hand, there have been few reports on the water activation effect on the ORR in alkaline media, although it is essential for the rational design of highly active alkaline ORR electrocatalysts.

It is well known that 3d transition metals usually have high water activation abilities^29^,^30^. Among various 3d transition metals, Mn has been reported to be particularly efficient for water activation owing to its extremely high oxophilicity for the adsorption of OH on its surfaces^28^. Therefore, in this study, we intended to utilize this high oxophilicity of Mn for water activation, and to prepare highly active Ag-based electrocatalysts for the ORR in alkaline media. In particular, we prepared a La perovskite oxide containing Mn (LaMnO_3_) and employed it as a Ag electrocatalyst support, because this structure could provide sufficient electrical conductivity to minimize Ohmic loss and act as an effective co-catalyst, as reported previously^31^,^32^.

Ag/LaMnO_3_ was synthesized through the simple loading of Ag nanoparticles prepared through the conventional colloidal method on LaMnO_3_ supports. The morphology of the prepared Ag/LaMnO_3_ was observed by transmission electron microscopy (TEM), and the crystal structure was identified through X-ray diffraction (XRD) measurements followed by Rietveld refinement with Fullprof. For the evaluation of the activity for the ORR in alkaline media, electrochemical methods such as the rotating ring-disk electrode (RRDE) method and cyclic voltammetry (CV) were employed. The variations in electronic structure of Ag and LaMnO_3_ were investigated to identify the ligand effect through X-ray photoelectron spectroscopy (XPS), electron energy loss spectroscopy (EELS), and density functional theory (DFT) calculations. Finally, the strain exerted on Ag on LaMnO_3_ was examined by using the extended X-ray absorption fine structure (EXAFS) data obtained in the synchrotron facilities.

## Results

**Figure 1:**
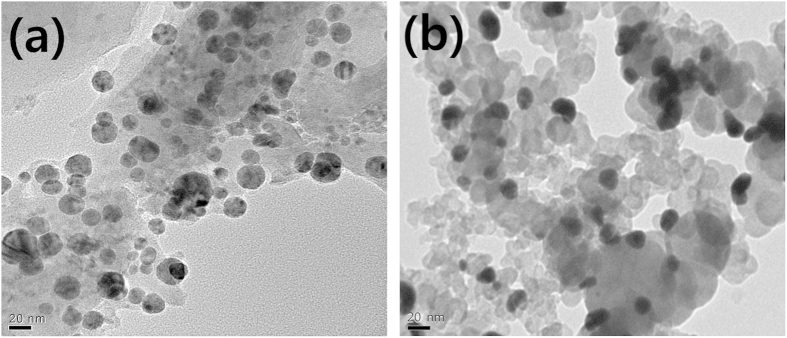
TEM micrographs of (a) Ag/LaMnO_3_ and (b) Ag/C.

The morphology of Ag synthesized with the citrate-protecting method and loaded on LaMnO_3_ and carbon supports, respectively, was observed by TEM, as shown in Fig. 1. In the TEM images, the dark spheres represent Ag particles, while the rather pale and larger particles correspond to the supports (LaMnO_3_ and carbon). The average particle size of Ag on carbon supports is smaller than 20 nm, while that on LaMnO_3_ supports of which the size is about 100 nm (see Figure S1) is about 20–30 nm. The larger average particle size for Ag on LaMnO_3_ might be therefore rather disadvantageous for the ORR activity. In general, since oxide particles synthesized through the coprecipitation process have few pores and relatively larger particle size, they are considered to be less advantageous as supports for the high dispersion of catalyst particles than porous carbon materials. With this reason, most previous studies on the metal oxide effect as co-catalysts on ORR activity also used carbon supports, because it was difficult to obtain a high dispersion on only oxide materials as supports^24^,^25^. However, in these cases, it could not be secured that Ag particles were loaded solely on oxide supports, so that it was hard to understand the exact effect of the metal and metal oxide interaction on the ORR activity. However, in this study since the Ag particles were loaded on only the perovskite oxide, a more precise investigation on the bifunctional effect at the interface between Ag and LaManO_3_ on ORR activity is possible. In particular, in order to clearly distinguish the interface effect at the direct contact between Ag and LaMnO_3_ from the simple additive effect of LaMnO_3_ into Ag/C, we prepared and examined Ag/C + LaMnO_3_ as well as Ag/LaMnO_3_.

**Figure 2:**
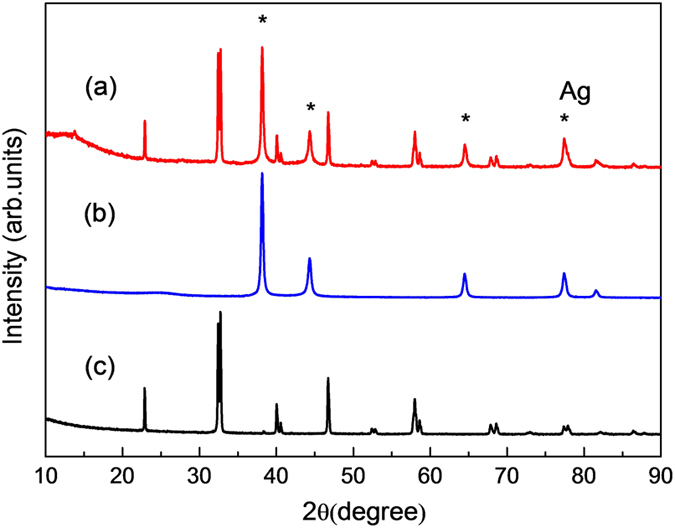
XRD patterns of (a) Ag/LaMnO_3_, (b) Ag/C, and (c) LaMnO_3_.

The crystal structures of prepared Ag particles and perovskite supports were examined through XRD techniques. Figure 2 presents the XRD patterns for Ag/LaMnO_3_, Ag/C, and LaMnO_3_. It was clearly identified through Rietveld refinement on the obtained XRD patterns that LaMnO_3_ was synthesized successfully as a single phase, showing the exact pattern corresponding to the perovskite (ABO_3_) structure without any side peaks. The crystal structure of Ag on both the LaMnO_3_ and carbon supports was also found to match exactly with the crystal index for the face-centered cubic (fcc) structure (2 *θ* = 38.3°, 44.2°, 64.4°, 77.4°, corresponding to the (111), (200), (220), and (311) facets, respectively)^21^. The XRD patterns for Ag/LaFeO_3_ and Ag/LaCoO_3_, which were prepared for comparison with Ag/LaMnO_3_ in view of the oxophilicity of B-site metal also well matched with the crystal index (see Figure S2). Table 1 and Figure S3 show the details of the results of Rietveld refinement of the XRD patterns. The crystal structure of LaMnO_3_ was found to be the rhombohedral unit cell (SG.167 R-3c) in a single phase, and was in good agreement with previous literature^33^,^34^. In addition, the structures of LaFeO_3_ and LaCoO_3_ were found to be the orthorhombic unit cell (SG.62 Pbnm) and the rhombohedral unit cell (SG.167 R-3c) in the single phase, respectively, as shown in Figure S3 and Table S1. All the lattice parameters and atomic positions obtained from the Rietveld refinement were employed as initial conditions for the DFT calculations of their electronic structures.

**Figure 3:**
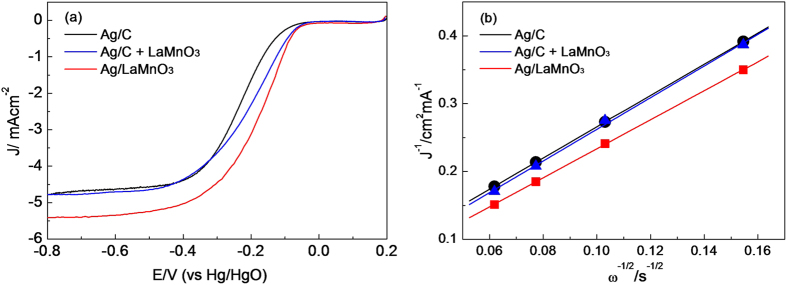
Oxygen reduction polarization curves for Ag/C, Ag/C + LaMnO_3_ and Ag/LaMnO_3_ at 1600 rpm in O_2_-saturated 0.1 M KOH at 10 mV s^−1^, and (b) Koutecky–Levich plots of the ORR for Ag/C, Ag/C + LaMnO_3_ and Ag/LaMnO_3_.

The ORR activity was examined through the well-established thin-film rotating disk electrode (TFRDE) techniques^35^. As shown in Fig. 3, the ORR activity for Ag/LaMnO_3_ measured at 1600 rpm was markedly higher than that for Ag/C. While the onset potential for ORR of Ag/C was about −0.1 V (vs. Hg/HgO), in good accordance with the data reported previously^6^,^21^, that for Ag/LaMnO_3_ was about 50 mV higher, implying that Ag/LaMnO_3_ have a high ORR activity than Ag/C. In particular, the limiting current for Ag/LaMnO_3_ was about 5.5 mA/cm^2^, which was close to that for Pt/C (5.62 mA/cm^2^), showing the nearly perfect four-electron pathway^24^, while that for Ag/C was below 5 mA/cm^2^, indicating that a considerable portion of ORR progressed via two-electron pathway leading to peroxide formation. It is interesting to note here that the mixture of Ag/C and LaMnO_3_ showed a clearly different behavior from Ag/LaMnO_3_. The onset potential for Ag/C + LaMnO_3_ was closed to that for Ag/LaMnO_3_, while their limiting current was rather similar to that for Ag/C. That is, the ORR kinetics for Ag/C + LaMnO_3_ is closed to that for Ag/LaMnO_3_, while the peroxide formation behavior was rather similar to Ag/C. This indicates that a special interaction between Ag and LaMnO_3_ could act as a key role in the different electrocatalytic behavior of Ag/LaMnO_3_ for ORR from Ag/C or Ag/C + LaMnO_3_.

In order to further understand the peroxide formation behavior via the exact calculation of the electron transfer number in the ORR, a Levich plot was drawn using the following Koutecky–Levich equation^36^, as shown in Fig. 3(b):


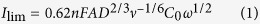


where *I*_lim_ is the limiting current density, *n* is the electron transfer number per oxygen molecule, *F* is the Faraday constant (*F* = 96485.3399 C/mol), *A* is the geometric area of the electrode (*A* = 0.19625 cm^2^), *D* is the diffusion coefficient of oxygen in 0.1 M KOH (*D* = 1.9 × 10^−5^ cm^2^/s), 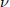 is the kinematic viscosity (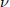 = 1.1 × 10^−2^ cm^2^/s), *C*_0_ is the oxygen concentration (*C*_0_ = 1.2 × 10^−6^ mol/cm^3^), and *ω* is the rotation speed in radians. It is widely recognized that the electrochemical reduction of oxygen has two pathways: the two-electron pathway to produce H_2_O_2_, and the direct four-electron pathway to produce H_2_O^21^. There is a consensus that the electron transfer number in the ORR for Pt/C is close to 4, leading to the direct formation of water^37^. The electron transfer number for Ag/C and Ag/C + LaMnO_3_ calculated with the Koutecky–Levich equation (the diffusion-controlled region, here E = −0.6 V was taken) was about 3.71 and 3.75, respectively, while that for Ag/LaMnO_3_ was about 3.98. This indicates that in the case of Ag/LaMnO_3_ the ORR predominantly proceeded via the four-electron pathway. Hence, it was clearly confirmed that the ORR activity of Ag/LaMnO_3_ with less peroxide formation was markedly higher than that of Ag/C or Ag/C + LaMnO_3_ and there is a special interaction between Ag and LaMnO_3_ which could be exclusively obtained from the Ag/LaMnO_3_ (Ag directly supported on LaMnO_3_) rather than the Ag/C + LaMnO_3_ mixture.

Because a specific activity is dependent on active site, electrochemical active surface area (ECSA) for each of the catalysts was compared. Figure S4 shows the CV curves for Ag/LaMnO_3_ and Ag/C. The electrochemical active surface area (ECSA) of the catalysts was determined by the AgO reduction peak of the CV. The AgO reduction peaks for Ag/LaMnO_3_ and Ag/C presented the value of 54.2 mC/cm^2^_geo_ and 59.3 mC/cm^2^_geo_, respectively. It can be therefore readily expected that since Ag/C have more active sites than Ag/LaMnO_3_, they are rather advantageous for ORR activity. However, as can be seen in the ORR polarization curves Ag/LaMnO_3_ demonstrated rather higher ORR activity than Ag/C, implying that the size effect cannot provide any reasonable discussion on the enhancement of ORR activity for Ag/LaMnO_3_. In order to fully understand the origin of the enhanced activity for Ag/LaMnO_3_, we intensively investigated three other major effects determining the ORR activity than the size effect: the bifunctional, ligand, and strain effects, in the following chapters.

## Discussion

It is widely recognized that the supply of protons to reduce O_2_ to H_2_O is relatively straightforward in acidic media as they are originally present there, while they must be generated by water activation in alkaline media^26^,^27^. For this reason, the water activation process is a significant step in determining the overall ORR kinetics in alkaline media. N. M. Markovic *et al.* demonstrated that in alkaline media, the combination of noble metal catalysts and 3d metal hydr(oxy)oxide co-catalysts having high oxophilicity led to a markedly enhanced electrocatalytic performance due to the bifunctional effect between metal and hydr(oxy)oxide^27^,^28^. Among various 3d transition metals, Mn has been widely recognized to be the most oxophilic component^27^,^30^. In particular, the oxophilicity of Mn can be increased further by combination with Ag, if there is a charge transfer between Mn and Ag. Hence, we anticipated that the Ag loading on LaMnO_3_ could result in a further increase in the oxophilicity of Mn, and therefore a higher water activation ability. In order to investigate the bifunctional effect in the ORR combined with water activation, we carefully reviewed the ORR polarization curves (Fig. 3(a)) obtained with the RRDE techniques.

The first notable point in the ORR polarization curves is that the onset potential was higher for both Ag/LaMnO_3_ and Ag/C + LaMnO_3_ than for Ag/C. In general, the ORR activity can be presented as the following formula^38^:


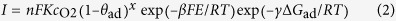


where *i* is the measured current density, *n* is the number of electrons, *K* is the rate constant, *x* is the order of active sites, *β* and *γ* are symmetry factors (assumed to be ½), *R* is the universal gas constant, *T* is the temperature, *c*_O2_ is the concentration of O_2_, *θ*_ad_ is the total surface coverage by adsorbed spectator species, and *G*_ad_ is the Gibbs free energy of reactive intermediates. There is a consensus that among various variants in the above formula, the key parameter determining the ORR kinetics is the (1–*θ*_ad_) term in many cases (representatively, the Pt surface), because the facile decrease in surface OH coverage can lead to an increase in active sites for the ORR^38^. However, in the case of Ag, this term does not affect the ORR activity at all, because surface hydroxide formation starts at a potential at least 0.2 V higher than the ORR termination (about −0.1 V vs. Hg/HgO). Hence, to find the origin of the enhanced ORR activity for Ag/LaMnO_3_, the reaction mechanism should be examined more carefully. It is well known that there are two pathways for the ORR in alkaline media, as follows^39^,^40^:

Direct four-electron pathway:


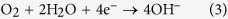


Two-electron pathway:


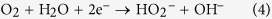



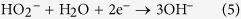


As shown in the above reaction pathways, a distinguished point in the ORR in alkaline media from that in acidic environments is that the water participates in the reaction not as a spectator but as a reactant, and therefore should be activated to progress the ORR. Thus, it is reasonable to infer that the ORR in alkaline media is terminated by the lack of water activation above around −0.1 V vs. Hg/HgO on Ag surfaces. If this hypothesis is proper, the origin of the higher onset potential for Ag/LaMnO_3_ or Ag/C + LaMnO_3_ is attributable to the high water-activation ability of LaMnO_3_. In the case of Ag/C, the water should be activated solely on the Ag surface, because the carbon surface is too inert to activate water. Furthermore, since Ag is also one of the noble metals with lower oxophilicity, the water activation cannot proceed efficiently. On the other hand, because LaMnO_3_ having a high oxophilicity can readily activate water, it can progress the ORR efficiently through the supply of protons to adsorbed oxygen molecules on the Ag surface. Water activation of LaMnO_3_ was confirmed by *in situ* XANES of Mn k-edge on LaMnO_3,_ as shown in Figure S5. Hence, it can be concluded that the origin of the higher onset potential for Ag/LaMnO_3_ or Ag/C + LaMnO_3_ is the bifunctional effect due to the water activation on LaMnO_3_.

**Figure 4:**
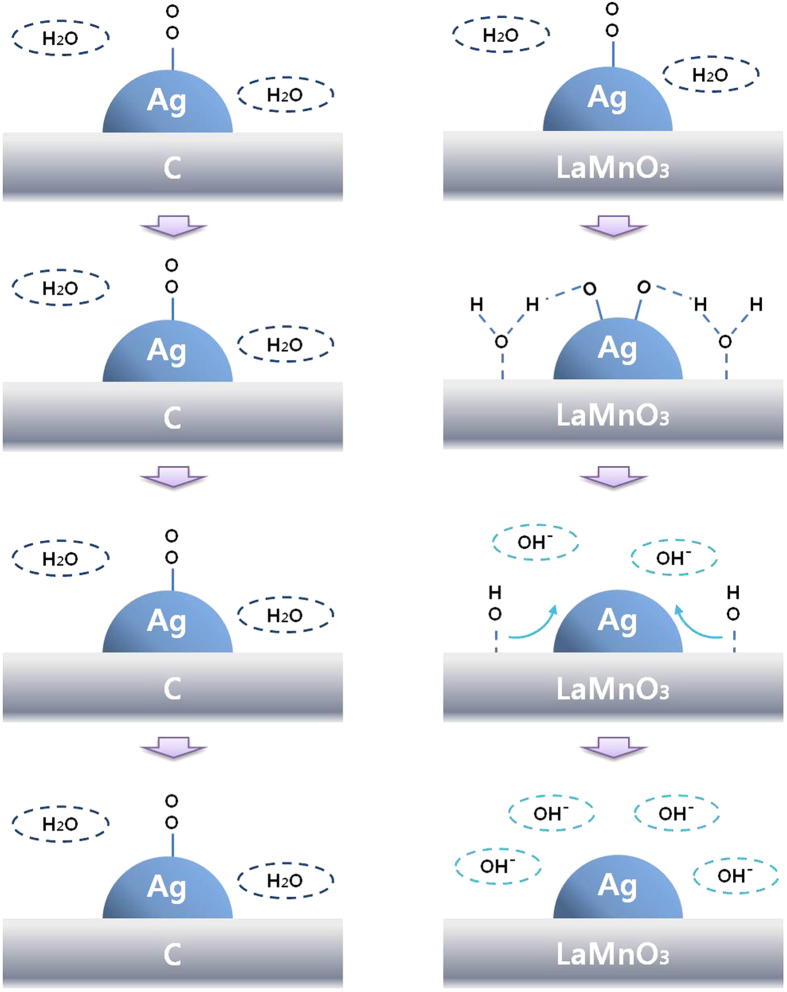
ORR scheme at around −0.1 V vs. Hg/HgO for Ag/C and Ag/LaMnO_3_.

The above discussion was demonstrated with a reaction scheme at around −0.1 V (vs. Hg/HgO), where Ag/C is already inactive, while Ag/LaMnO_3_ is still active for the ORR, as shown in Fig. 4. In the case of Ag/C, the adsorbed oxygen on the Ag surface cannot be activated for the ORR, because neither Ag nor carbon can activate water to provide protons to the adsorbed oxygen at this potential. On the other hand, in the case of Ag/LaMnO_3_ or Ag/C + LaMnO_3_ the adsorbed oxygen on the Ag surfaces can be effectively activated by the proton supply to the adsorbed oxygen on Ag via water activation on LaMnO_3_ at this potential.

The effect of water activation on LaMnO_3_ was also found in the CV curves as shown in Figure S4. A notable point in the CV curves is that the oxidation of Ag/LaMnO_3_ starts at lower potential than Ag/C, implying that the surface of Ag/LaMnO_3_ is oxidized more readily than that of Ag/C. It has been reported that the OH spillover from support to metal leads to facile oxide formation on the metal surface, which changes the catalytic performance^41^. In particular, the hydroxyl spillover is prominent in metal/metal oxide composite catalysts containing 3d transition metals owing to the strong metal support interaction (SMSI)^42^,^43^. Since the oxidation of silver should be initiated by water activation on the Ag surface, the hydroxyl spillover from co-catalysts can be a great help in proceeding the Ag surface oxidation. Hence, the lower oxidation potential for Ag/LaMnO_3_ which might be attributed to the hydroxyl spillover can also support the bifunctional effect due to water activation on LaMnO_3_.

The effect of oxophilicity on the water activation was reconfirmed through the complementary experiments in which the main variation was the B-site metal (M = Mn, Fe, Co) in perovskite materials. It has been already reported that since the oxophilicity can be varied with the kind of metal (for example, the order of oxophilicity is Co < Fe < Mn), the variation of the B-site with such metals in the perovskite materials can be effective for the examination of the role of oxophilicity on the ORR activity in alkaline media^27^. Figure S6 shows the kinetics-controlled region in the ORR polarization curve with the Tafel plot, which is advantageous for comparison of the onset potentials. As expected, the order of onset potential (Ag/LaCoO_3_ < Ag/LaFeO_3_ < Ag/LaMnO_3_) was consistent with that of the oxophilicity. Hence, it is clearly confirmed that the employment of materials with high oxophilicity to promote the water activation as co-catalysts or supports can enhance the ORR activity in alkaline media.

**Figure 5:**
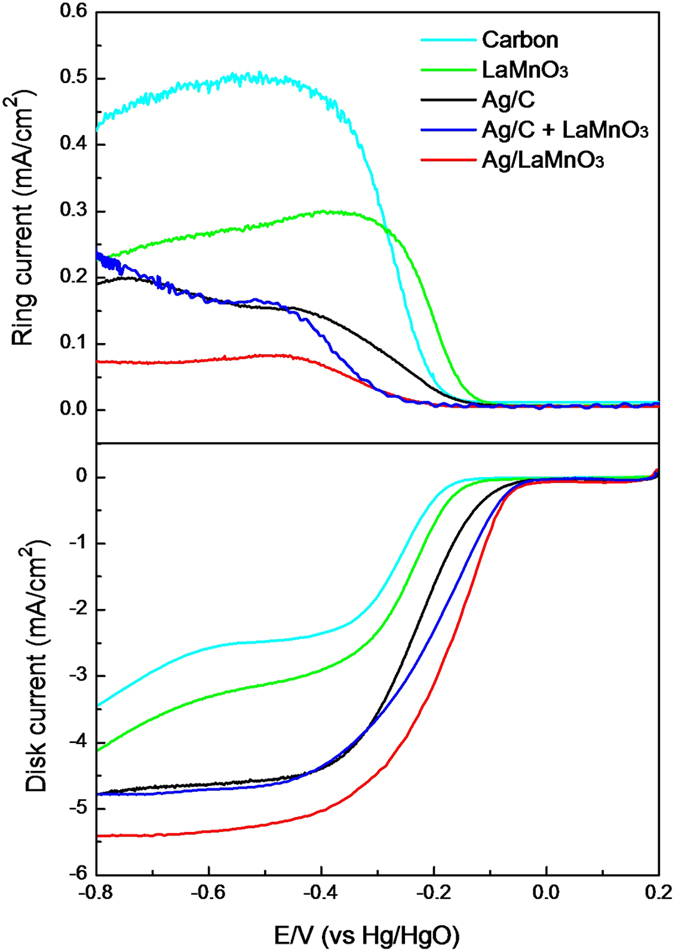
RRDE measurements of the ORR for carbon, LaMnO_3_, Ag/C, Ag/C + LaMnO_3_ and Ag/LaMnO_3_ at 1600 rpm in O_2_-saturated 0.1 M KOH at 10 mV s^−1^. Collection efficiency *N* = 0.37; ring potential *E*_r_ = 0.3 V vs. Hg/HgO.

The second notable point in the ORR polarization curves is that the limiting current density of Ag/LaMnO_3_ is exclusively closed to the theoretical value for the four-electron pathway, while that of Ag/C or Ag/C + LaMnO_3_ is lower than the value. This indicates that the ORR on Ag/C or Ag/C + LaMnO_3_ include a considerable portion of peroxide formation (two-electron pathway). In particular, the peroxide formation behavior of Ag/C + LaMnO_3_ is rather similar to Ag/C than Ag/LaMnO_3_ as opposed to the case of onset potential where Ag/C + LaMnO_3_ is rather closed to Ag/LaMnO_3_ than Ag/C. In order to understand the different behavior on the peroxide formation on various electrocatalysts, we employed the RRDE techniques with a fixed potential in the ring electrode to detect the peroxide formed. Figure 5 shows the polarization curves of the disk and ring electrode for five kinds of samples: Ag/LaMnO_3_, Ag/C + LaMnO_3_, Ag/C, LaMnO_3_, and carbon supports. Since we used the electrode having same geometric surface area in the RRDE experimental setup for all the samples, the limiting current should be same under the assumption that all the samples have enough kinetics for ORR. However, the order of limiting current density was carbon <LaMnO_3_ <Ag/C <Ag/C + LaMnO_3_ <Ag/LaMnO_3_ and that for Ag/LaMnO_3_ was exclusively closed to the theoretical value calculated by Levich equation. This indicates that all the samples except for Ag/LaMnO_3_ have an insufficient activity to progress the ORR via the direct four-electron pathway in alkaline media. As shown in the polarization curves on the ring electrode in Fig. 5, it is clearly demonstrated that Ag/C + LaMnO_3_, Ag/C, LaMnO_3_, and carbon showed very high ring current densities owing to the peroxide oxidation, implying that a high portion of the ORR proceeds via the two-electron pathway. The peroxide formation for Ag/C and carbon is well explained by the insufficient water activation on Ag and carbon, and that for LaMnO_3_ is attributable to the sluggish oxygen activation due to its high oxophilicity. On the contrary, in the case of Ag/LaMnO_3_ since there is a synergetic bifunctional effect originating from the high water activation on LaMnO_3_ and the high oxygen activation on Ag, it demonstrates a markedly enhanced ORR activity in alkaline media. It should be noted here that even though Ag/C + LaMnO_3_ can also meet the criteria for ORR activity enhancement (the high water activation on LaMnO_3_ and the high oxygen activation on Ag), the peroxide formation on them is much higher than that on Ag/LaMnO_3_. The Ag particles in Ag/LaMnO_3_ are directly attached on the LaMnO_3_, while those in Ag/C + LaMnO_3_ are directly supported on carbon and have limited contacts with LaMnO_3_ in the simple mixture. Hence, it can be concluded that the interface between Ag and LaMnO_3_ is very critical to determine the peroxide formation behavior and should be maximized to obtain the best ORR activity in alkaline media.

The variation of onset potential for peroxide formation in the ring current can also support our discussion on the bifucntional effect. While the onset potential for carbon changed little after Ag loading in Ag/C, that for LaMnO_3_ shifted significantly to a more negative potential in Ag/LaMnO_3_. This result also clearly demonstrated the synergetic bifunctional effect in Ag/LaMnO_3_. In the case of Ag/C, as there is no synergetic effect between Ag and the carbon support, peroxide formation is progressed separately on both Ag and carbon. Thus, even though the total amount of peroxide formation was reduced by loading Ag, the onset potential remained the same. Little synergy effect was also shown in Ag/C + LaMnO_3_, where the Ag particles have very limited contacts with LaMnO_3_ in the simple mixture. On the other hand, a marked shift of the onset potential for peroxide oxidation was shown for Ag/LaMnO_3_. The peroxide formation on the LaMnO_3_ surface is attributable mainly to insufficient oxygen activation due to the high oxophilicity. The marked shift in the onset potential for peroxide oxidation therefore indicates that the oxygen activation ability was significantly enhanced after Ag loading. It can therefore be inferred that in the case of Ag/LaMnO_3_, the oxygen activation was progressed mainly on the Ag surface, and the LaMnO_3_ just played the role of co-catalyst to assist the water activation.

**Figure 6:**
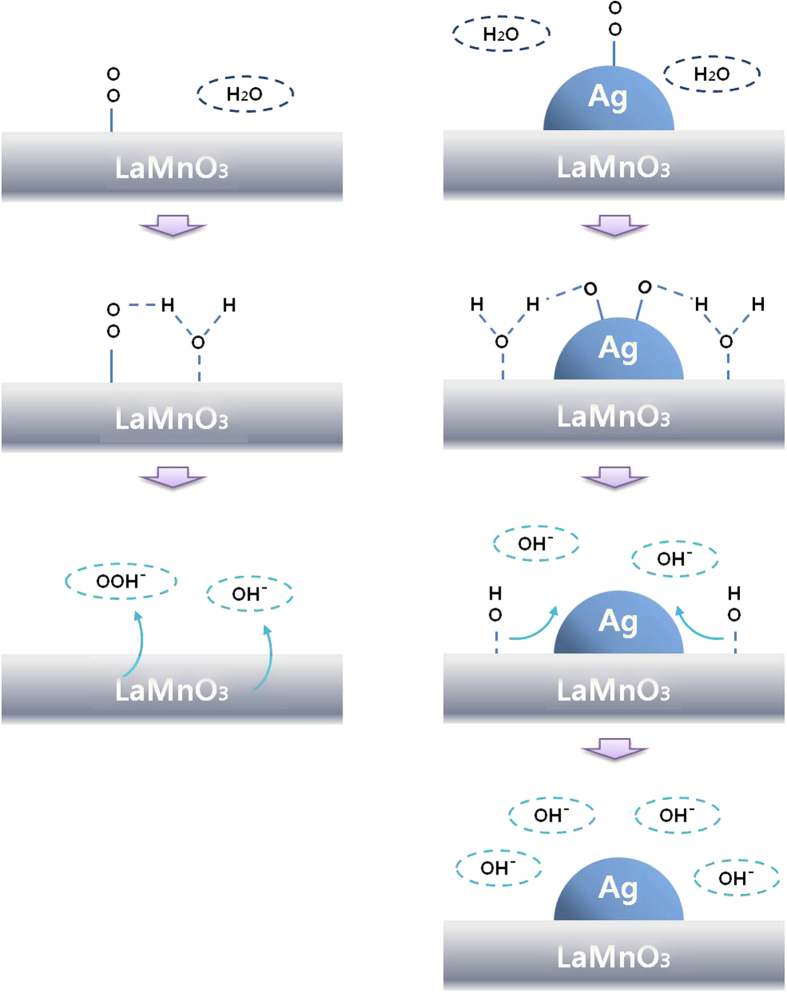
ORR scheme at around −0.2 V vs. Hg/HgO for LaMnO_3_ and Ag/LaMnO_3_.

The possible ORR scheme mentioned above is rendered in Fig. 6. At around −0.2 V vs. Hg/HgO, where peroxide formation is progressed on LaMnO_3_ but not on Ag/LaMnO_3_, the bifunctional effect can effectively suppress the formation of HO_2_^−^ on the LaMnO_3_ surface. For this reason, the onset potential for peroxide oxidation at the ring electrode could be shifted markedly to a more negative potential. Hence, this lower peroxide formation on Ag/LaMnO_3_ due to the synergetic bifunctional effect could lead to a marked enhancement of the ORR activity in alkaline media.

### Ligand effect

Another major factor determining the activity for the ORR is the electronic effect to induce a perturbation in the electronic structure, which can have a direct effect on the adsorption strength of oxygen species on the surface of an electrocatalyst^44^,^45^. The electronic effect can be categorized into two major types: the ligand effect due to the charge transfer between catalyst metals and other components in co-catalysts or supports, and the strain effect due to the lattice strain caused by the difference in atomic radius or the interaction with heterogeneous atoms. For an in-depth examination of the electronic structure change in Ag/LaMnO_3_, we performed XPS (for the occupied state), EELS (for the unoccupied state) measurements and DFT calculations for the ligand effect, and EXAFS analyses with the synchrotron beam for the strain effect.

**Figure 7:**
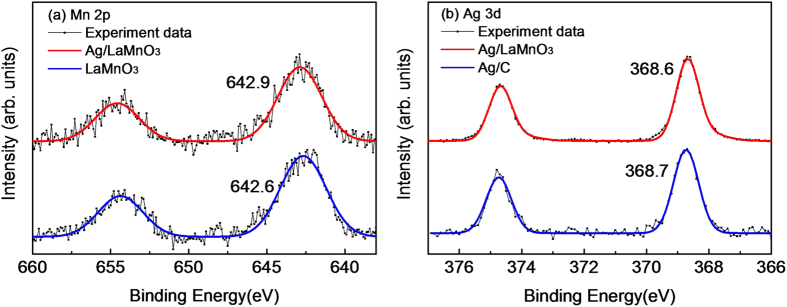
XPS for (a) Mn 2p of Ag/LaMnO_3_ and LaMnO_3_, and (b) Ag 3d of Ag/LaMnO_3_ and Ag/C.

**Figure 8:**
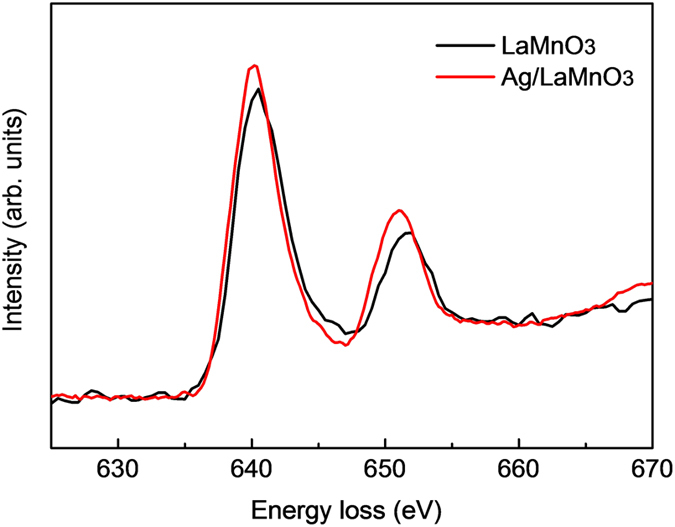
Mn L_2,3_ edge EELS obtained from Ag/LaMnO_3_ and LaMnO_3_.

The XPS spectra for the Mn 2p and Ag 3d core levels are presented in Fig. 7. The binding energies of the Mn 2p_3/2_ orbital were measured to be 642.6 and 642.9 eV for LaMnO_3_ and Ag/LaMnO_3_, respectively, as shown in Fig. 7(a). The obtained binding energy (642.6 eV) for LaMnO_3_ is consistent with the values for Mn(III) reported in previous literature^46^, while that for Ag/LaMnO_3_ (642.9 eV) is located between Mn(III) and Mn(IV), for which the binding energy is 645 eV. Such a blueshift to higher binding energy after Ag loading on LaMnO_3_ indicates that the charge transfer occurred from Mn to Ag because of the work function difference between Ag and LaMnO_3_. The binding energies of the Ag 3d_5/2_ core orbitals were measured to be 368.7 eV (FWHM = 0.86) and 368.6 eV (FWHM = 0.95) for Ag/C and Ag/LaMnO_3_, respectively, as shown in Fig. 7(b). This result is in good agreement with the previous report that the FWHM for Ag supported on the oxide was greater than that for Ag on carbon by the peak broadening due to the charge transfer^47^. The slight redshift of the binding energy for Ag/LaMnO_3_ in comparison with Ag/C implies that Ag/LaMnO_3_ is in a slightly more electron-rich phase than Ag/C because of the charge transfer from Mn, as already ascertained from the XPS results on the Mn 2p_3/2_ core level. This charge transfer to Ag was shown consistently for other perovskite supports used in this study, such as Ag/LaCoO_3_ and Ag/LaFeO_3_, as shown in Figure S7. To reconfirm the charge transfer in the aspect of the unoccupied state, we performed EELS analyses. Figure 8 shows the EELS spectra for the Mn L_2_ (2p_3/2_ → 3d) edge and L_3_ (2p_1/2_ → 3d) edge of LaMnO_3_ and Ag/LaMnO_3_. The results clearly show an increase in peak intensity after Ag loading on LaMnO_3_, implying that the d-band vacancy of Mn was increased by the charge transfer from Mn to Ag.

Even though the XPS and EELS studies can provide evidence of charge transfer, in-depth information on the electronic structure, such as the electron population for each orbital or the total charge in each component cannot be obtained. We therefore employed DFT calculations with the CASTEP code^48^ based on a plane-wave expansion of the wave functions and a Vanderbilt-type ultrasoft pseudopotential formalism^49^. Table 2 shows the calculated Mulliken electronic populations and total charges for LaMnO_3_, Ag/LaMnO_3_, and Ag/C. The difference in total charge clearly reconfirms that the charge transfer occurs from Mn to Ag, and that the Ag on LaMnO_3_ is in an electron-rich state in comparison with Ag on carbon. Notably, in the results of DFT calculations, the charge difference in Ag between LaMnO_3_ and carbon is much more dominant in the sp-band than the d-band, implying that the charge transferred from Mn is located principally in the sp-band rather than the d-band of Ag. S. Linic *et al.* reported that an increase in electronic population in the sp-band can strengthen the adsorption of oxygen species owing to the decrease in repulsion^50^. Hence, it is believed that the adsorption strength of oxygen species is higher for Ag/LaMnO_3_ than for Ag/C. As Norskov *et al.* suggested^44^,^45^, the adsorption strength of oxygen species on Ag surfaces is weaker than the optimal strength for the ORR. The marked increase in total charge in the sp-band of Ag/LaMnO_3_ may therefore be advantageous in terms of the ORR activity. In addition to the change in the Ag electronic state, the decrease in the total charge in Mn sites may lead to an increase in oxophilicity, which will enhance the water-activation ability of LaMnO_3_. The charge transfer can therefore result in a positive effect on both the oxygen activation on Ag and the water activation on Ag/LaMnO_3_. The variation of the Mulliken electronic population on LaFeO_3_, LaCoO_3_, and Ag/LaCoO_3_ was also calculated, and showed exactly the same trend as Ag/LaMnO_3_ (see Table S2). However, in the case of Ag/LaCoO_3_, since the total charge change in Ag is less notable than that in Ag/LaMnO_3_, the ligand effect on the ORR activity could be less effective.

### Strain effect

**Figure 9:**
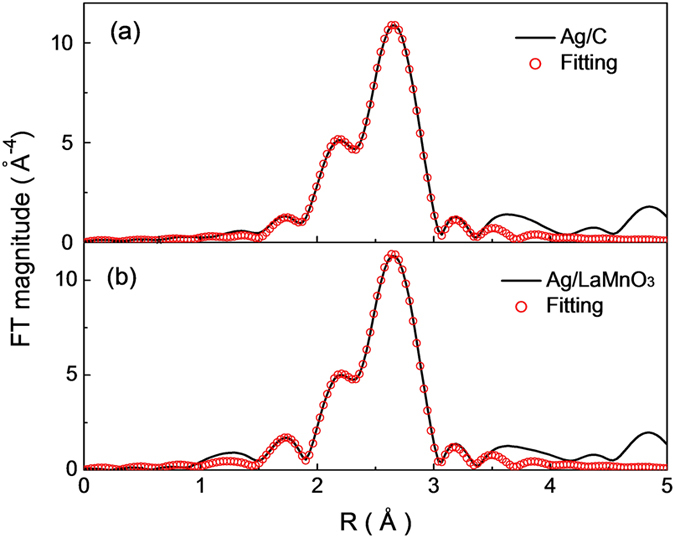
Fourier transforms of EXAFS spectra at the Ag K-edge of (a) Ag/C and (b) Ag/LaMnO_3_.

In addition to charge transfer, the lattice strain can also cause a great change in the electronic structure of electrocatalysts; this is known as the strain effect. To examine the effect of lattice strain in Ag/LaMnO_3_, we employed EXAFS analysis, which is a promising tool for understanding the fine structure in a lattice. Figure 9 and Table 3 show the Fourier-transformed radial distribution functions and the data fitting results to the theoretical model calculated with the FEFF 8.3 code for Ag/LaMnO_3_ and Ag/C, respectively. Figure S8 shows the XAFS Ag *K*-edge raw data of the XAFS and *k*^*2*^ weighted EXAFS data of Ag/C and Ag/LaMnO_3_. The bond length of 1NN Ag–Ag was measured to be 2.85 Å for Ag/LaMnO_3_ and 2.86 Å for Ag/C. Even though the bond length for Ag/LaMnO_3_ is slightly shorter, it is reasonable not to consider this small difference as physically meaningful. This is because the EXAFS data fitting results are obtained through multistep complex mathematical treatment processes, which can broaden the margin of error. It is widely recognized that the bond length decrease originates from the lattice compressive strain^51^. In our previous studies, we demonstrated that there is a great lattice compressive strain in Pt nanoparticles supported on Ti-based oxide materials, and this is the main origin of the enhanced ORR activity^52^. The strong metal support interaction (SMSI) induced between Pt and Ti sites led to an increase in contact area between them, and therefore to an increase in surface tension. However, the lattice strain is negligible in the case of Ag/LaMnO_3_ because Ag has a much weaker d–d interaction owing to the fully filled d-band (d^10^-state), and the particle size is too large (about 20–30 nm) to induce lattice strain through interaction with the support. Hence, it is believed that the strain effect in Ag/LaMnO_3_ is negligible for the enhancement of the ORR activity.

## Conclusion

In this study, we have examined the effect of water activation on the ORR in alkaline media. Ag supported on LaMnO_3_ having a high water-activation ability owing to their highly oxophilic nature demonstrated a markedly enhanced ORR activity in comparison with Ag/C. In order to understand the origin of this activity enhancement, we have investigated three major effects in detail: bifunctional, ligand, and strain effects induced by Ag loading on LaMnO_3_. In terms of the bifunctional effect, the origin of the enhanced ORR activity was revealed to be the synergetic combination of the high water activation on LaMnO_3_ and the high oxygen activation on Ag. Furthermore, such bifunctional effect was found in exclusively at the direct contact between Ag and LaMnO_3_ in Ag/LaMnO_3_, not in the indirect mixture of Ag/C + LaMnO_3_. From the perspective of the ligand effect, it was manifested that the charge transfer to Ag from Mn strengthened the adsorption of oxygen species on Ag and increased the oxophilicity of the Mn sites, which positively affected the ORR activity. On the other hand, the strain effect was negligible, because the d–d interaction in Ag was too weak and the particle size of Ag was too large to induce lattice strain. In summary, the origin of the markedly enhanced ORR activity for Ag/LaMnO_3_ in alkaline media is therefore attributable to the bifunctional effect due to water activation on LaMnO_3_ having high oxophilicity, partly supported by the ligand effect. On the basis of these results, we suggest that the water-activation ability should be considered as one of the most critical factors in the design of ORR electrocatalysts in alkaline media. Hence, it is essential to employ co-catalysts or supports with a highly oxophilic nature and to maximize the interface between catalyst and support.

## Methods

### Synthesis

The coprecipitation process was used to synthesize LaMnO_3_^53^,^54^. La(CH_3_COO)_3_ • 1.5H_2_O and (99.9%, Alfa Aesar), Mn(CH_3_COO)_2_ • 4H_2_O (≥99%, Sigma-Aldrich), in the respective stoichiometric ratio, were mixed in deionized (DI) water at metal concentrations in the order of 0.03 M. The solution was stirred for more than 24 h at room temperature. The metal source was fully dissolved in the solution, which was evaporated in a vacuum evaporator, and dried at 80 °C in an oven for 24 h. Finally, the precursor was obtained. This precursor was annealed at 1000 °C for 24 h to form LaMnO_3_. After the heat treatment, the LaMnO_3_ powder was washed with DI water. The powder was dehydrated using a centrifuge and dried at 80 °C in an oven to give the product. LaFeO_3_ and LaCoO_3_ particles were synthesized with the same method for LaMnO_3_ using Fe(CH_3_COO)_3_ • 9H_2_O (≥99%, Sigma-Aldrich) and Co(CH_3_COO)_2_ • 4H_2_O (≥99%, Sigma-Aldrich) respectively. Silver loadings on LaMnO_3_ particles of 40 wt% were achieved through the citrate-protecting method^55^. AgNO_3_ (0.127 g, 99.9+%, Alfa Aesar) and sodium citrate (0.5 g, 99%, Alfa Aesar) were dissolved in DI water (100 mL), and NaBH_4_ (0.01 g) was also dissolved in DI water (10 mL). Then, the NaBH4 solution was added dropwise under vigorous stirring to obtain a yellowish-brown Ag colloid. Subsequently, LaMnO_3_ (0.12 g) was dispersed in DI water (50 mL), which was then ultrasonicated for 1 h and added to the Ag colloid as the support. After one day, the suspension was filtered, washed with a copious amount of DI water, and dried in an oven at 60 °C. Ag/C, Ag/LaFeO_3_ and Ag/LaCoO_3_ particles were synthesized with the same method for Ag/LaMnO_3_.

### Characterization

The morphology of the powder was observed with a transmission electron microscope (TEM, Jem 2011) in the Korea Basic Science Institute (KBSI). The powder X-ray diffraction (XRD) analysis was performed with an Advance X-ray diffractometer (D8 ADVANCE) with CuK_α_ radiation (*λ* = 1.54056 Å). X-ray diffraction data were collected over the 2*θ* range 10–90° with a step size of 0.02° and a counting time of 0.2 s per step. The data were analyzed by the Rietveld analysis method. Fullprof software was employed for retained XRD Rietveld refinement. X-ray photoelectron spectroscopy (XPS) analysis was performed with an ESCALAB 250 instrument in the Korea Basic Science Institute (KBSI), and electron energy loss spectroscopy (EELS) analysis was performed with a high-resolution transmission electron microscope (HR TEM) in the Korea Institute of Science and Technology (KIST). Extended X-ray absorption fine structure (EXAFS) data at the Ag-K edge (25516.5 eV) were obtained in transmission mode with the synchrotron radiation of wide X-ray absorption fine structure spectroscopy (10C beamline, Pohang Light Source (PLS)) at room temperature. The spectra were processed using the program IFEFFIT^56^,^57^ (version 1.2.11, IFEFFIT, Copyright 2008, Matthew Newville, University of Chicago, http://cars9.uchicago.edu/ifeffit/) with background subtraction (AUTOBK) and normalization. Fourier transform for EXAFS spectra was performed in the range 2.5–12 Å in *k*-space and 1–3.25 Å in *R*-space with the first-shell single scattering paths and k^3^ weighting.

### Electrochemical measurements

The catalyst (10 mg) and acetylene black (AB) carbon (3 mg, 99.9+%, Alfa Aesar) were mixed with RDE solution (2 mL), as reported previously^35^,^58^ for the preparation of a working electrode. Acetylene black carbon was used to eliminate any concerns regarding electronic conductivity limitations^35^. After homogenization by sonication, 20 μL of the ink was drop-cast onto a glassy carbon electrode and then evaporated at room temperature to form a catalyst thin film. The rotating disk electrode (RDE) measurements were performed in a three-electrode electrochemical cell with a potentiostat (Biologic VSP) at room temperature. The working electrode (AFE3T050GC, Pine) was a catalyst-film-covered glassy carbon disk (0.19625 cm^2^ in area). A Pt wire and a mercury/mercury oxide (Hg/HgO) electrode were used as the counter and reference electrodes, respectively. Oxygen reduction reaction (ORR) measurements were performed in the potential range between −0.8 and 0.2 V(vs. Hg/HgO) in an oxygen-saturated 0.1 M KOH aqueous solution at rotation rates of 100, 400, 900, 1600, and 2500 rpm at a scan rate of 10 mV/s. The rotating ring-disk electrode (RRDE) measurements were performed at room temperature. The working electrode (AFE7R9GCPT, Pine) was a catalyst-film-covered glassy carbon disk (0.2475 cm^2^ in area), and the ring electrode was polycrystalline Pt (0.1866 cm^2^ in area). The collection efficiency of the RRDE was *N* = 0.37, and the potential of the ring electrode was held at 0.3 V during the RRDE measurements.

### Calculations

In this work, we performed DFT calculations using the Cambridge Sequential Total Energy Package (CASTEP) code of Materials Studio^48^, and used a plane-wave expansion of the wavefunctions and a Vanderbilt-type ultrasoft pseudopotential formalism^49^. We used the exchange-correlation functional based on the generalized gradient approximation (GGA) in the Perdew–Burke–Eruzerhof (PBE) scheme. LaMnO_3_ has the R-3C structure (space group No. 167), which has two representations: hexagonal and rhombohedral. In our calculations, the hexagonal unit cell containing 30 atoms was relaxed, and all the Mn atoms were at the centers of the MnO_6_ octahedra. We also added eight Ag atoms on the (012) surface of LaMnO_3_ to perform calculations on Ag/LaMnO_3_. The Ag 4d5s electrons, La 5s5p5d electrons, Mn 3d4s electrons, and O 2s2p electrons were treated as the valence states. The plane-wave cutoff energy was 380 eV, and converged results were achieved with a (3 × 5 × 2) *k*-point mesh.

## Additional Information

**How to cite this article**: Park, S.-A. *et al.* Bifunctional enhancement of oxygen reduction reaction activity on Ag catalysts due to water activation on LaMnO_3_ supports in alkaline media. *Sci. Rep.* **5**, 13552; doi: 10.1038/srep13552 (2015).

## Supplementary Material

Supplementary Information

## Figures and Tables

**Table 1:** Rietveld refined structural parameters of LaMnO_3_.

Atom position	x	y	z	B/Å^2^
LaMnO_3_				
space group R-3c (No.167)				
a = 5.5265(1) b = 5.5265(1) c = 13.3545(1)				
Rp = 4.34 Rwp = 5.62 Rexp = 4.51 S = 1.25				
La	0.0	0.0	0.25	0.3
Mn	0.0	0.0	0.0	0.3
O	0.444(2)	0.0	0.25	0.3

Note. Isotropic thermal parameters (B) are fixed to be 0.3 Å^2^.

**Table 2:** The electron occupied states for s, p, d, and f and the calculated charge transfer of LaMnO_3_, Ag/LaMnO_3_, and Ag/C.

Element		s	p	d	f	q_i_
LaMnO_3_	La	2.110	6.160	1.210	0	1.520
	Mn	0.350	0.530	5.620	0	0.490
	O	1.850	4.820	0	0	−0.670
Ag/LaMnO_3_	Ag	0.912	0.398	9.872	0	−0.181
	La	2.068	6.101	1.252	0	1.578
	Mn	0.330	0.448	5.618	0	0.600
	O	1.857	4.804	0	0	−0.665
Ag/C	Ag	0.818	0.183	9.862	0	0.136
	C	1.169	2.865	0	0	−0.033

**Table 3:** Curve fitting results of the EXAFS data at Ag K-edge of the catalysts.

	shell	N	R(Å)	σ^2^(Å)
Ag/C	Ag-Ag	9.6	2.86(1)	0.01
Ag/LaMnO_3_	Ag-Ag	10.1	2.85(1)	0.01
