# Supplementary material for: Bifunctional enhancement of oxygen reduction reaction activity on Ag catalysts due to water activation on LaMnO3 supports in alkaline media
Source: Sci Rep. 2015 Aug 27;5:13552. doi: 10.1038/srep13552 (PMC4550837; doi:10.1038/srep13552)
Supplement: Supplementary Information [file srep13552-s1.docx]

**Bifunctional enhancement of oxygen reduction reaction activity on Ag catalysts due to water activation on LaMnO_3_ supports in alkaline media**

***Shin-Ae Park^1^, Eun-Kyung Lee^1^, Hannah Song^1^, and Yong-Tae Kim*^1^***

*^1^Department of Energy System, Pusan National University, Busan 609-735, Republic of Korea.*

**Correspondence and requests for materials should be addressed to Y.-T.K. (email: yongtae@pusan.ac.kr).*

**Supporting Information**


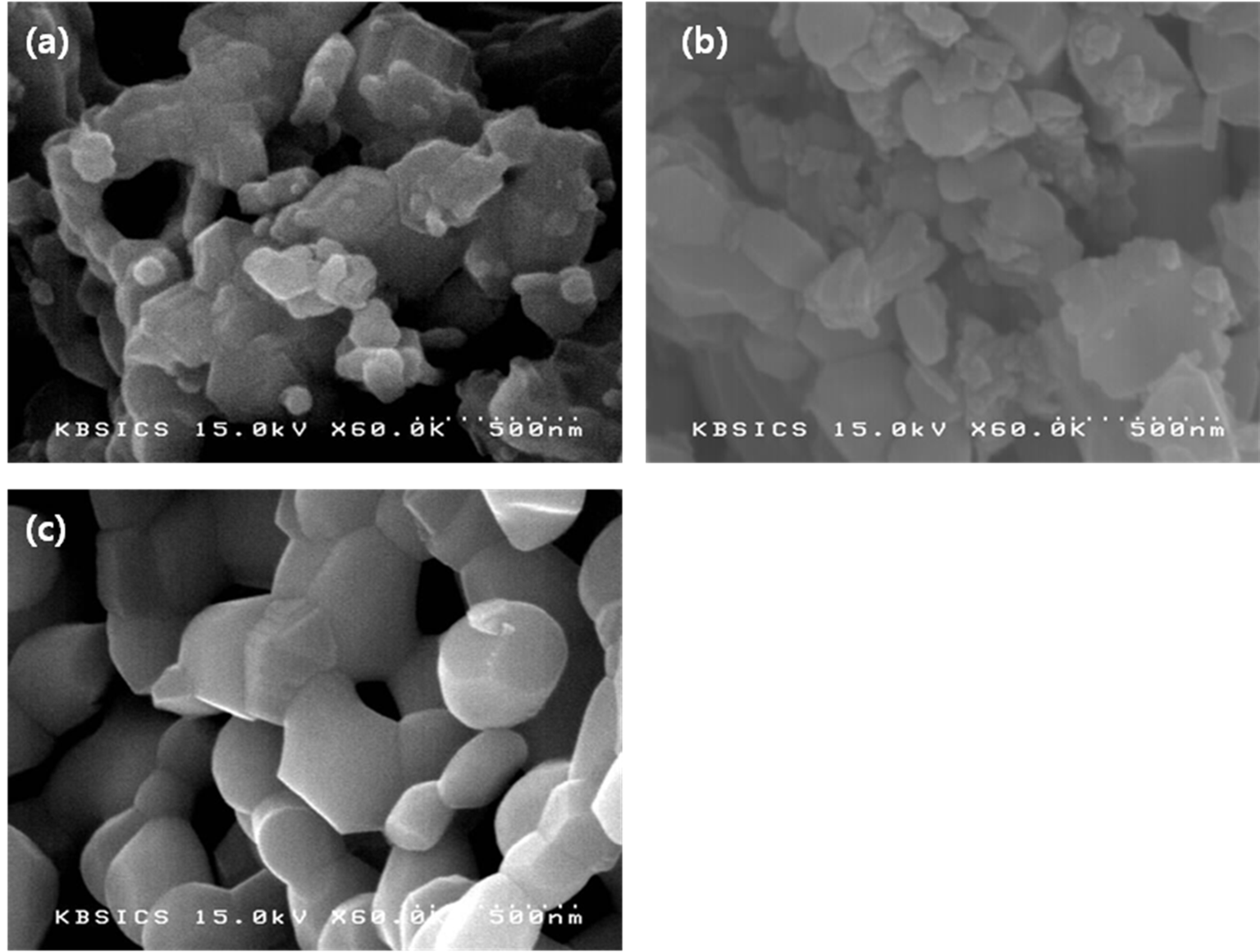


**Figure S1**. SEM images of (a) LaMnO_3_, (b) LaFeO_3_, and (c) LaCoO_3_.


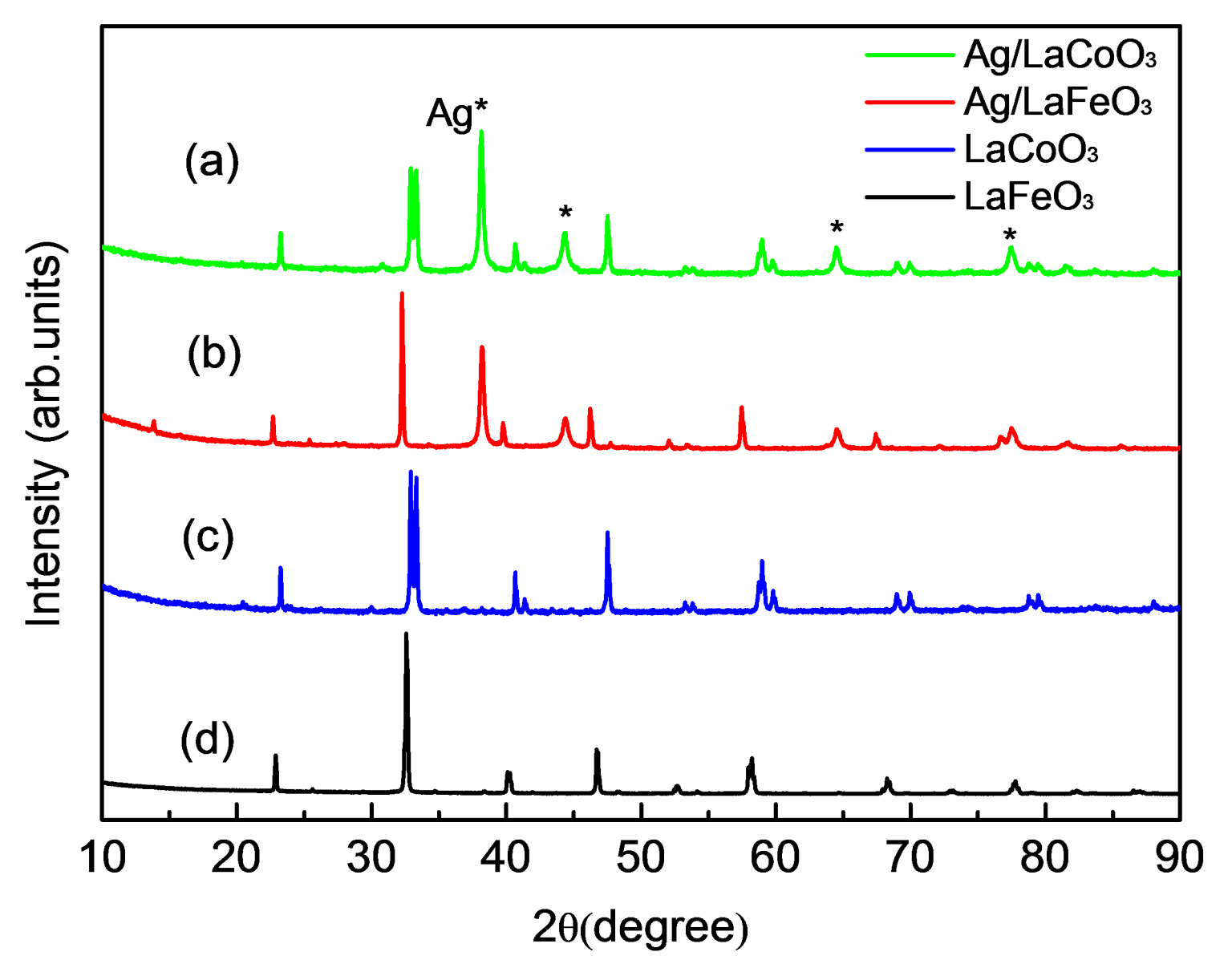


**Figure S2**. XRD patterns of (a) Ag/LaCoO_3_, (b) Ag/LaFeO_3_, (c) LaCoO_3_, (d) LaFeO_3_.


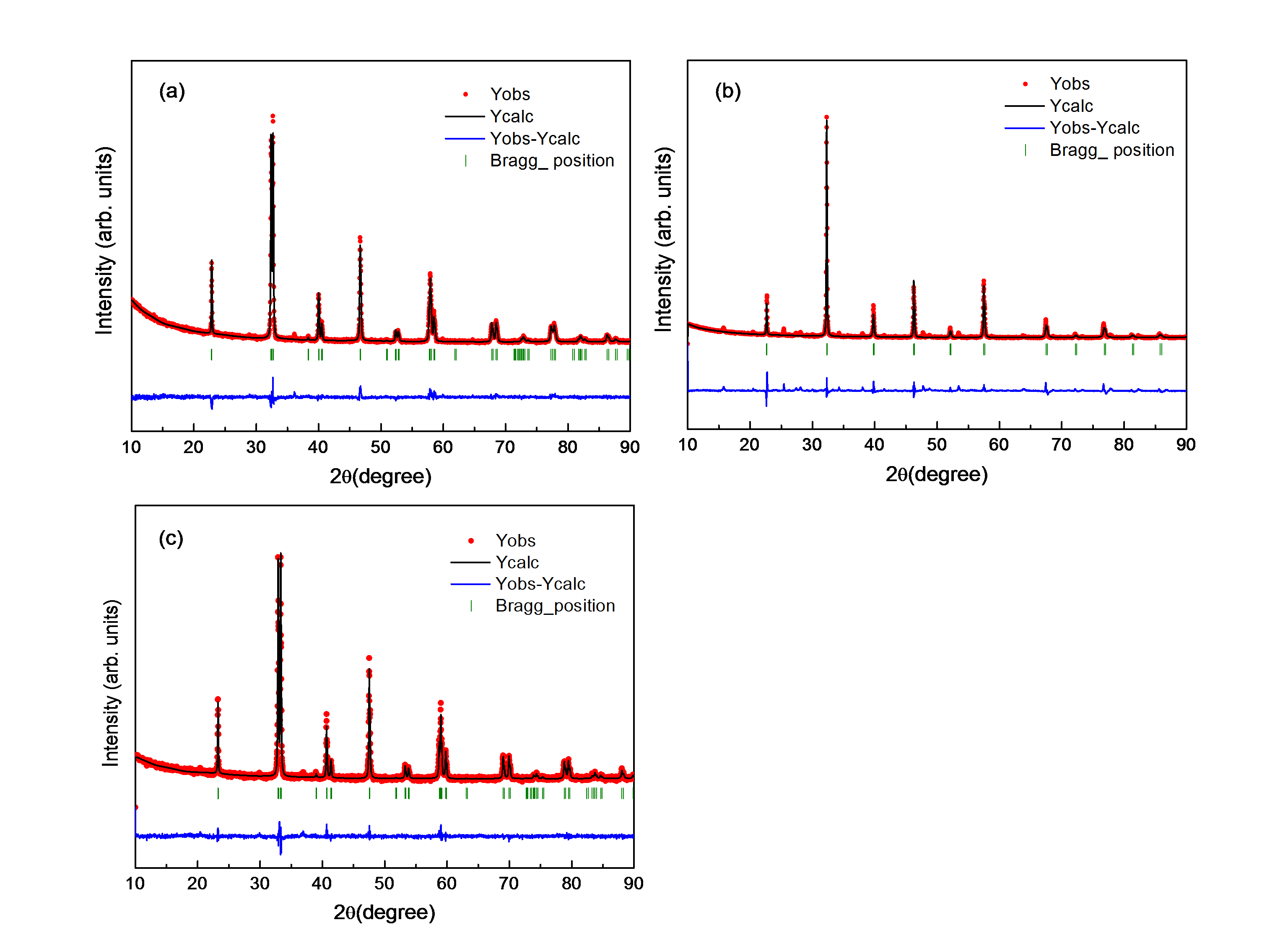


**Figure S3**. Rietveld refinement for (a) LaMnO_3_, (b) LaFeO_3_ and (c) LaCoO_3_.


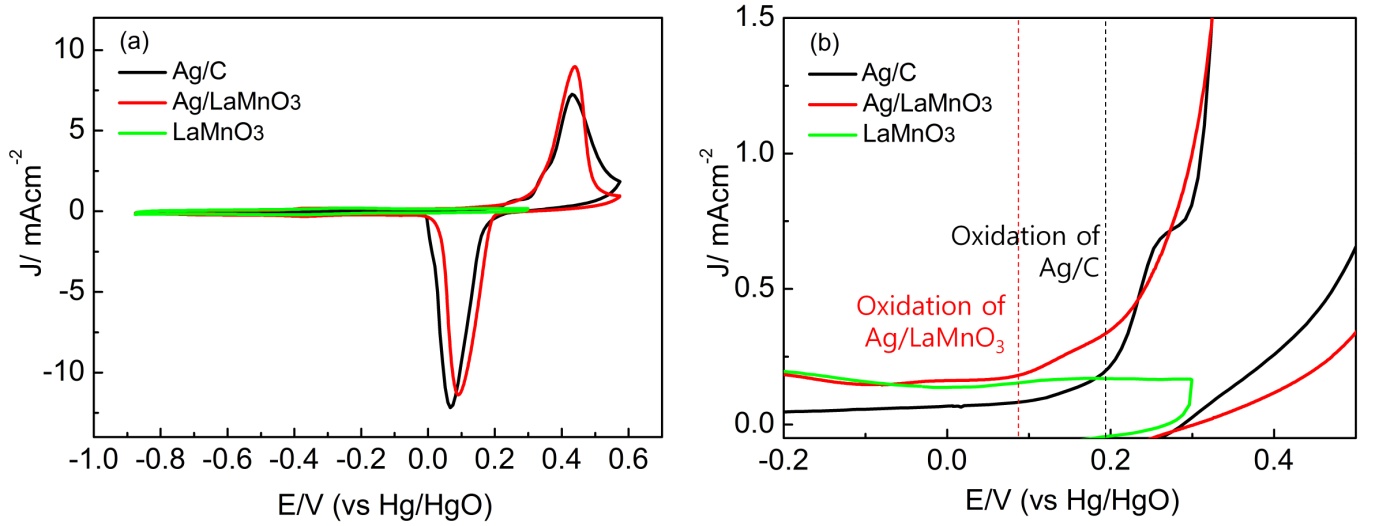


**Figure S4.** (a) Cyclic voltammetry of Ag/C, Ag/LaMnO_3_ and LaMnO_3_ catalysts in N_2_-purged 0.1 M KOH solution at 20 mV s^-1^ and (b) part of the graph (a) magnified.

**
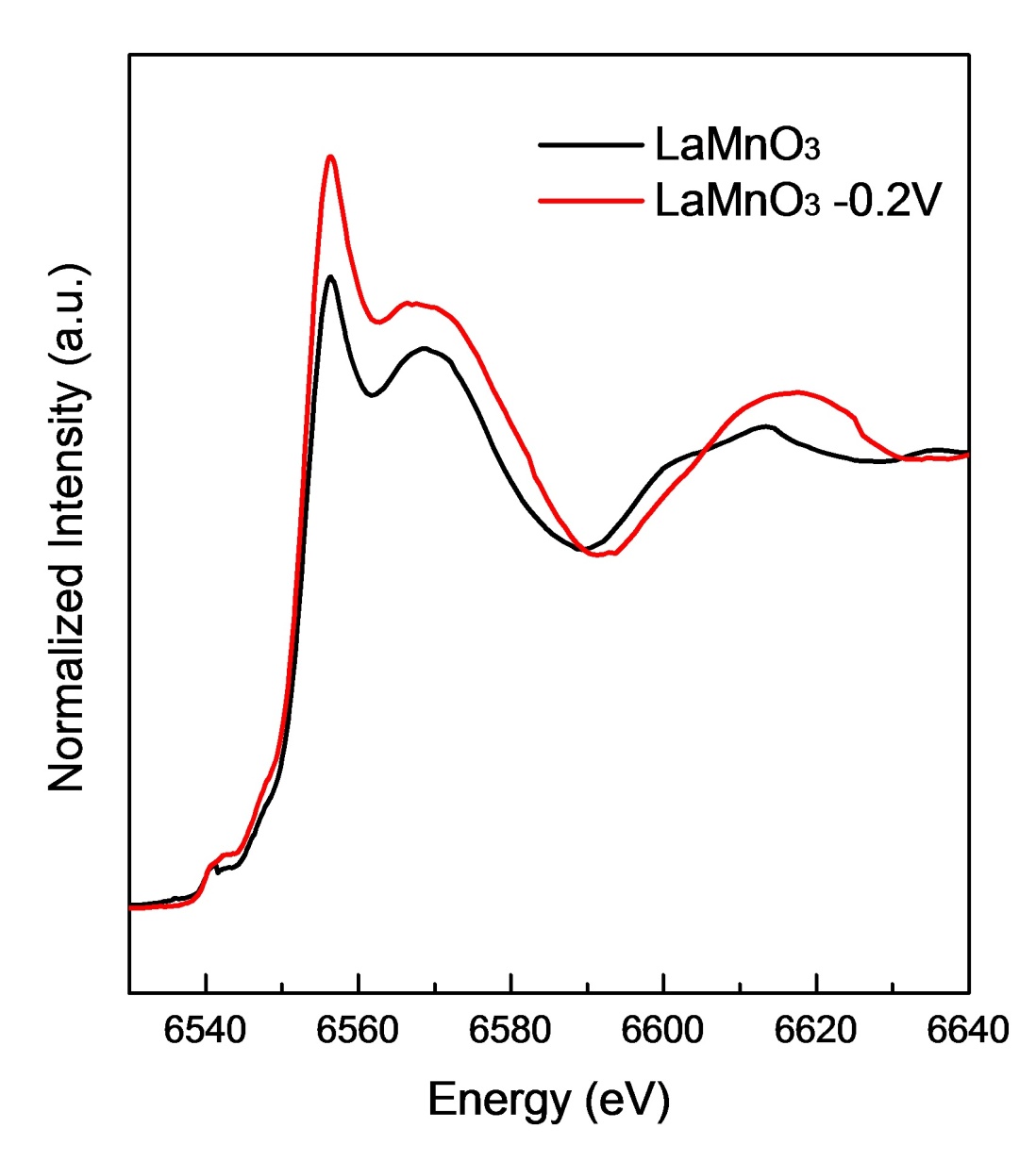
**

Figure S5. In situ XANES spectra of Mn k-edge with potential application on LaMnO_3_. The increase of the white line for LaMnO_3_(at -0.2V vs Hg/HgO) reveals oxidation of LaMnO_3_ by water activation.

**
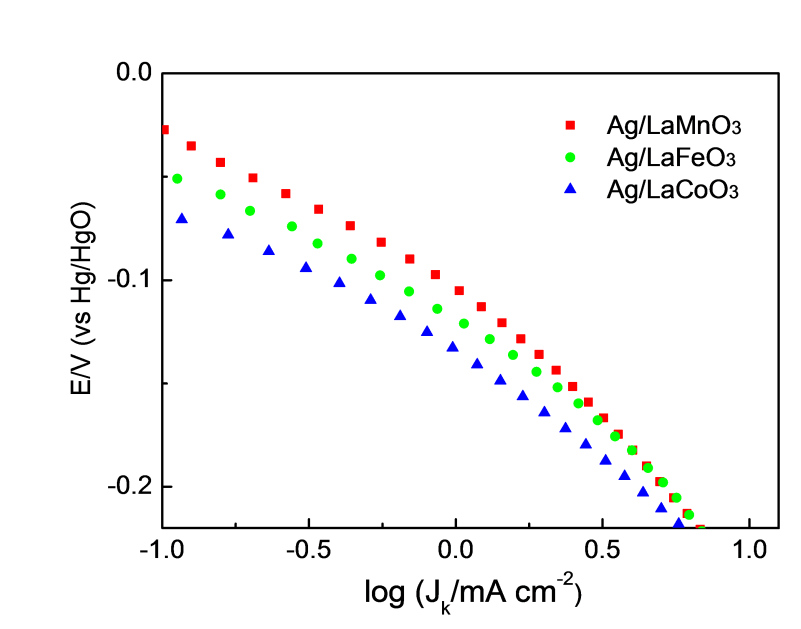
**

**Figure S6.** Tafel plots of the ORR for Ag/LaMO_3_ (M=Mn,Fe,Co) at 1600 rpm in O_2_-saturated 0.1 M KOH at 10 mV s^-1^.


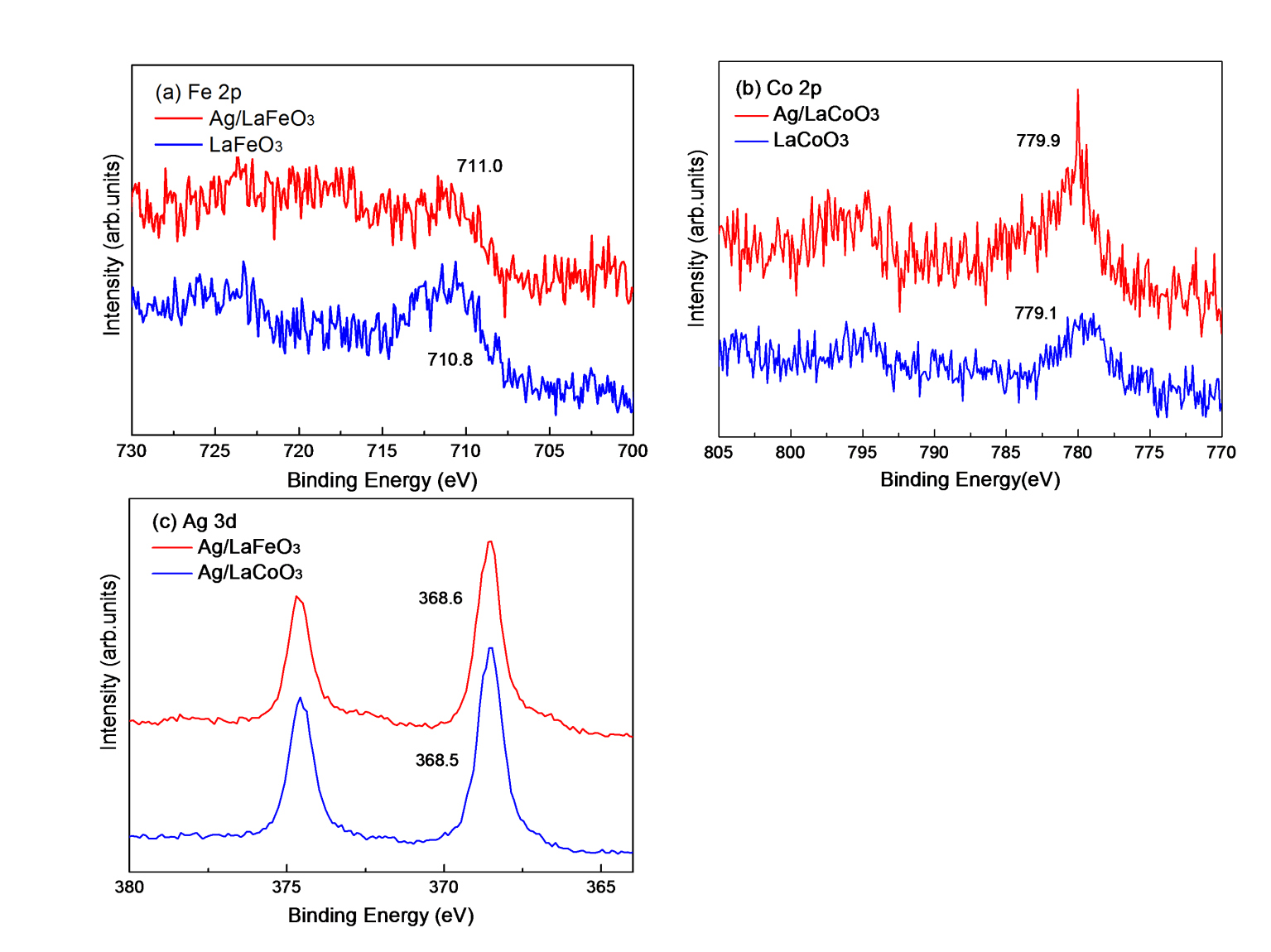


**Figure S7**. XPS for (a) Fe 2p of LaFeO_3_ and Ag/LaFeO_3_, (b) Co 2p of LaCoO_3_ and Ag/LaCoO_3_, and (c) Ag 3d of Ag/LaFeO_3_ and Ag/LaCoO_3_.


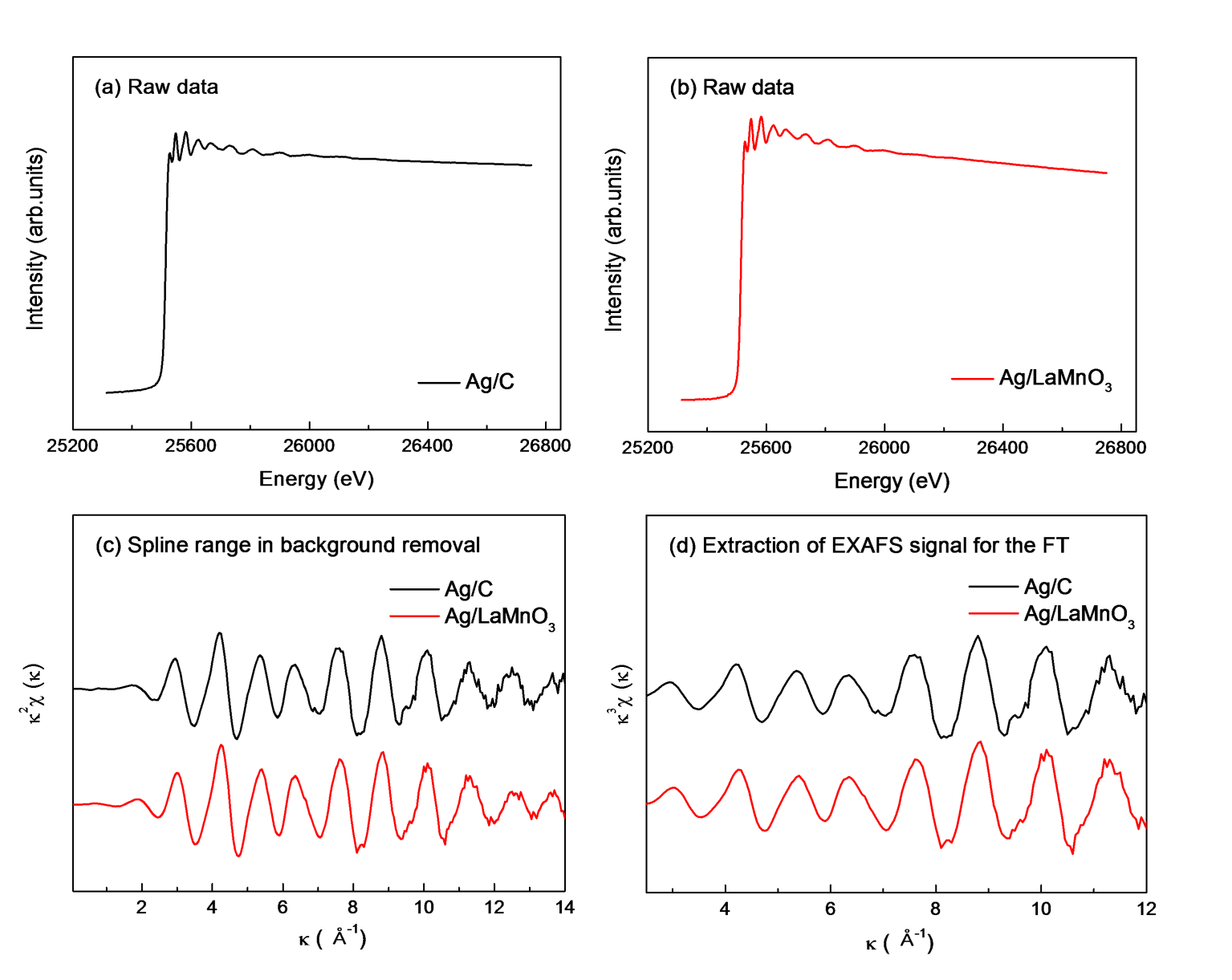


**Figure S8.** XAS Ag *K*-edge raw data of (a) Ag/C, (b) Ag/LaMnO_3_, (c) *k^2^* weighted EXAFS data of Ag/C and Ag/LaMnO_3_, which are represented in *k*-space with the spline range of 0.05 – 14 Å^-1^, and (d) k^3^ weighted EXAFS data of Ag/C and Ag/LaMnO_3_ for the FT with the range of 2.5 -12 Å^-1^. All XAS Pt *L_3_*-edge data for the calculation of Ag-Ag bond length were obtained in the transmission mode at the Pohang accelerator laboratory (PAL) using the 10C wide XAFS beam line. Data were collected from -200 eV to 1200 eV relative to the E_0_. Raw data were calibrated with the signal of Ag foil then normalized to the jumping of the absorption edge after the pre-edge range was set to zero. The final EXAFS data were obtained by performing a FT on the *r*-space, which was set to 1-3.25 Å after the extraction of EXAFS signal for the FT from *k*-space.

**Table S1.** Rietveld refined structural parameters of LaFeO_3_ and LaCoO_3_.

| Atom position | x | y | z | B/ Å^2^ |
| --- | --- | --- | --- | --- |
| LaFeO_3_  space group Pbnm (No.62)  a= 5.5852(1) b=5.5728(1) c=7.8709(1)  Rp=3.94 Rwp=5.14 Rexp=4.06 S=1.26 | | | | |
| La | 0.012(1) | 0.025(1) | 0.25 | 0.3 |
| Fe | 0.0 | 0.5 | 0.0 | 0.3 |
| O1 | -0.012(35) | 0.490(7) | 0.25 | 0.3 |
| O2 | -0.292(8) | 0.223(10) | 0.050(3) | 0.3 |
| LaCoO_3_  space group R-3c(No.167)  a= 5.4409(1) b=5.4409(1) c=13.0913(1)  Rp=2.65 Rwp=3.43 Rexp=2.66 S = 1.29 | | | | |
| La | 0.0 | 0.0 | 0.25 | 0.3 |
| Co | 0.0 | 0.0 | 0.0 | 0.3 |
| O | 0.551(3) | 0.0 | 0.25 | 0.3 |

Note. Isotropic thermal parameters (B) are fixed to *be 0.3 Å^2^*

**Table S2.** The electron occupied states for s, p, d, and f and the calculated charge transfer of LaFeO_3_, LaCoO_3_, and Ag/LaCoO_3_.

| Element |  | | s | p | d | f | q_i_ |
| --- | --- | --- | --- | --- | --- | --- | --- |
| LaFeO_3_ | | La | 2.100 | 6.100 | 1.200 | 0 | 1.590 |
|  |  | Fe | 0.390 | 0.690 | 6.480 | 0 | 0.460 |
|  |  | O | 1.850 | 4.820 | 0 | 0 | -0.680 |
| LaCoO_3_ | | La | 2.040 | 6.110 | 1.250 | 0 | 1.590 |
|  |  | Co | 0.450 | 0.650 | 7.420 | 0 | 0.490 |
|  |  | O | 1.840 | 4.850 | 0 | 0 | -0.690 |
| Ag/LaCoO_3_ | | Ag | 0.843 | 0.428 | 9.862 | 0 | -0.131 |
|  |  | La | 2.127 | 6.033 | 1.263 | 0 | 1.576 |
|  |  | Co | 0.398 | 0.531 | 7.425 | 0 | 0.647 |
|  |  | O | 1.869 | 4.830 | 0 | 0 | -0.698 |
